# Estimation of soil salt content in the Bosten Lake watershed, Northwest China based on a support vector machine model and optimal spectral indices

**DOI:** 10.1371/journal.pone.0273738

**Published:** 2023-02-24

**Authors:** Jiawen Hou, Yusufujiang Rusuli

**Affiliations:** 1 College of Geography and Remote sensing Sciences, Xinjian University, Ürümqi, 830046, People’s Republic of China; 2 Key Laboratory of Oasis Ecology Ministry of Education, Xinjian University, Ürümqi, 830046, People’s Republic of China; 3 Institute of Geography and tourism, Xinjiang Normal University, Ürümqi, 830054, People’s Republic of China; Shandong University, CHINA

## Abstract

Low-cost and efficient dynamic monitoring of surface salinization information is critical in arid and semi-arid regions, we conducted a remote sensing inversion exercise for soil salinity in the Bosten Lake watershed in Xinjiang, Northwest China, with a total area of about 43,930 km^2^, a typical watershed in an arid area. Sentinel MSI and Landsat OLI data were combined with measured soil salinity data in July 2020, and optimal combination bands were selected based on characteristic bands to create a grid search-support vector machine (GS-SVM) inversion model of soil salt content. The maximum value of soil salt content in the Bosten Lake watershed was 11.8 g/kg. The minimum value was 0.41 g/kg, and the average value was 4.77 g/kg, soil salinization is serious. The results of previous studies were applied to the estimation of salt content in Bosten Lake watershed and could not meet the monitoring requirements of the study area, *R*^2^ < 0.3. The GS-SVM soil salinity monitoring model was established based on the optimal DI, RI, and NDI remote sensing indexes for the Bosten Lake watershed. After model verification, it was found that the optimal model of image data was the Landsat OLI first-derivative model with *R*^2^ of 0.64, RMSE of 3.12, and RPD of 1.64, indicating that the prediction ability of the model was high. We used the first-order derivative model of Landsat OLI data to map the soil salt content in the Bosten Lake watershed in arid area, and found that soil salt content in most of the study area was between 10 and 20 g/kg, indicating severe salinization. This study not only reveals the distribution characteristics of salinization in Bosten Lake watershed, but also provides a scientific basis for soil salinization monitoring in Central Asia to lay a foundation for further soil salinization monitoring in arid areas.

## 1 Introduction

Soil salinization has become an important research issue in the construction of an ecological civilization and the monitoring of global change [1–3]. The low fertility of saline soil affects crop growth and hinders sustainable development of the agricultural economy. Long-term irrigation causes soil secondary salinization, especially in arid and semi-arid irrigated agricultural areas, which has a great impact on regional agricultural production and is associated with serious ecological environmental risks. Therefore, salinization monitoring and management are matters of wide concern [3–6]. To prevent soil salinization and rationally develop and use salinized land, it is necessary to study the physical and chemical properties of salinized soil and monitor their changes. Remote sensing, as an effective technique for soil salinity monitoring, has been widely used in soil salinity inversion and mapping [6,7]. Monitoring of the soil salt content is the key to scientific research on saline-alkali soil. In-depth mining of information on saline-alkali soil from remote sensing data is of great relevance for the effective treatment of saline-alkali soil, prevention of further degradation, the rational development and utilization of saline-alkali soil resources, and ensuring ecological sustainable development.

Remote sensing quantitative inversion involves establishing a specific model based on the relationship between pixel values in remote sensing images and the information retrieved from the corresponding ground points. These models can be used to accurately and practically describe surface information, but their monitoring accuracy needs to be further studied. The application of laboratory or space ground spectroscopy can accurately solve a large number of soil and related environmental problems, such as soil erosion, soil salinity, soil moisture content, or heavy metals [8,9]. There is a close relationship between the characteristics of the soil reflectance spectrum and the physical and chemical properties of soil in salinized areas, which provides a basis for the application of remote sensing technology to study soil properties [10]. Many studies have focused on distinguishing soil types and inverting soil organic matter, water, nitrogen, and heavy metal contents based on soil reflection spectral characteristics [11]. Hyperspectral remote sensing has a high spectral resolution, which can be used to quantitatively obtain soil biochemical components and estimate the soil salt content based on the soil reflection spectrum. Based on hyperspectral data, Liu et al. found that differences in soil moisture and salinity had a major impact on spectral characteristics, especially within each absorption zone of water (1400, 1900, and 2200 nm) [12]. Although there are many studies of soil salt content based on hyperspectral data, only a few have combined multispectral data with soil salt content modeling [13–15]. Bouaziz et al. established a linear spectral unmixing model for the estimation of soil salt content in northeastern Brazil using spectral parameters that affect salinization, such as the vegetation index, extracted from MODIS remote sensing images together with regression analysis [15]. Chen et al. constructed a support vector machine (SVM) model of soil salt content based on Landsat 8 OLI multi-spectral image and improved vegetation index with the 7th band, and obtained good inversion results for the spatial distribution of soil salt contents. Wang et al. and Zhu constructed an inversion model of soil salinization in Ebinhu Basin of Xinjiang based on machine learning model by using satellite multi-spectral data, achieving high-precision extraction of soil salinization in Ebinhu Basin and achieving a breakthrough of spectral index from two-dimensional to three-dimensional [16,17]. Han et al constructed two-dimensional soil salinization inversion index of Ebinhu River Basin in Xinjiang by using satellite multi-spectrum, realizing high-precision extraction of soil salinization of Ebinhu River Basin and achieving a breakthrough of spectral index from two-dimensional to three-dimensional [18]. Therefore, multi-spectral remote sensing data also have a high application value in salinization monitoring [19].

In this study, we examined the Bosten Lake watershed, a representative arid area, and applied combined Landsat OLI data and Sentinel MSI image data to explore the potential of previous spectral parameters for soil salinity inversion. A grid search-support vector machine (GS-SVR) regression model was then established to monitor the soil salt content in the Bosten Lake watershed by optimizing the spectral index. The specific research objectives were as follows: (1) to explore the universality of the optimal estimation parameters in previous studies in the arid Bosten Lake watershed, (2) to select the optimal estimation parameters and establish a GS-SVR regression model, and (3) to map soil salinity in the Bosten Lake watershed. The purpose of this study is to lay a foundation for soil salinization monitoring in arid areas and further provide a scientific basis for soil salinization monitoring in Central Asia.

## 2. Study area

### 2.1 Study area

**Fig 1:**
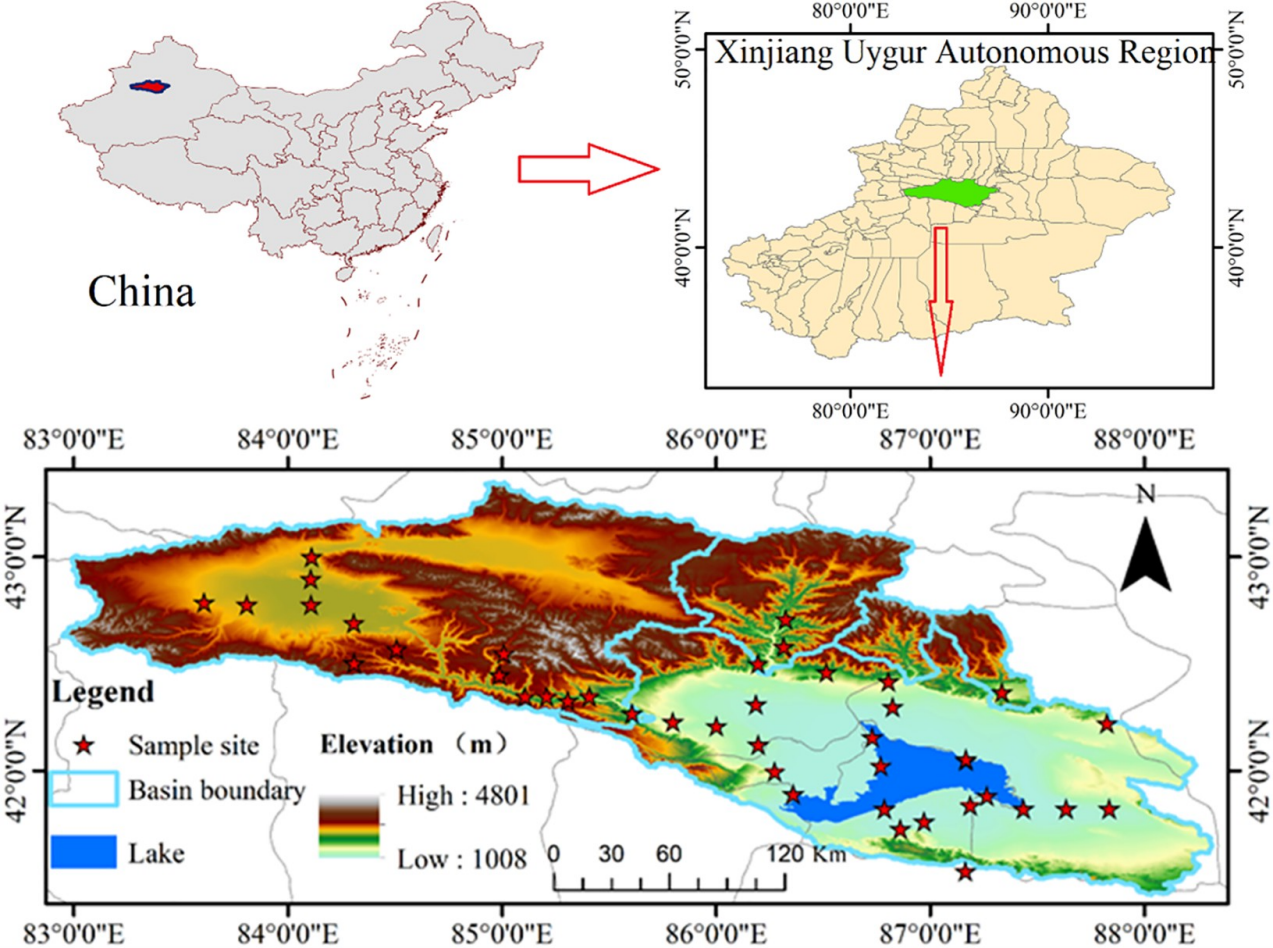
Map of the Bosten Lake watershed.

The Bosten Lake watershed (82°54′10″–88°21′06″E, 41°21′19″–43°21′34.8″N) is located in the Bayin’guoleng Mongol Autonomous Prefecture in Xinjiang, Northwest China, with a total area of about 43,930 km^2^ and elevations of 1,008–4,801 m. The regional terrain is generally high in the northwest and low in the southeast. The study area mainly consists of the watersheds of the Kaidu River (including the Great Youerdusi watershed and the Little Youerdusi watershed), the Huangshuigou River, the Qingshui River, the Ushtala River, and the Yanqi River, and more than 20 temporary river watersheds (Fig 1). The Bosten Lake watershed has a temperate continental arid climate, with a long sunshine duration, a mean annual evaporation of 2,368 mm, and a mean annual precipitation of only about 60 mm (more than 80% of which occurs in summer). The rivers in the watershed are mainly recharged by alpine snowmelt and ice-melt, as well as rainfall. The regional landscape varies with elevation mainly including, in order of decreasing elevation, glacial snow belt, meadow steppe belt, oasis plain, desert steppe belt, desert belt and the Bosten Lake. Bosten Lake is surrounded by mountains and has a typical continental desert climate, which is characterized by a dry spring with little precipitation, a dry and hot summer, a cool fall and a cold winter [20].

### 2.2 Soil data collection

Field surveys were conducted in the Bosten Lake watershed from July 13, 2020 to August 23, 2020. Specifically, surface soil samples were collected from 43 sites on the north and south shores of Bosten Lake, as well as in the Kaidu River watershed (Fig 1). The samples were transported back to the laboratory for analysis. The analysis process was as follows: (1) the soil samples were allow to air-dry indoors; (2) the air-dried samples were ground, crushed and passed through a 2-mm sieve; (3) 20 g of soil sample was weighed and mixed with 100 mL of deionized water (i.e., a water-soil ratio of 5:1, v/w) to form a soil slurry; and (4) the soil salt content was determined using a conductivity/salinity meter (Orion 115 A+, Thermo Fisher, USA), with a relative accuracy ±0.1 mV or 0.05% and the soil pH was measured using a pH electrode [21,22].

### 2.3 Remote sensing image data collection

Landsat-8 OLI and Sentinel-2 series remote sensing data with medium spatial resolution were selected. The imaging time was August 2020, which was in summer and had good weather in the study area to provide favorable conditions for the inversion of soil salt content. Specific imaging information is shown in Table 1. Landsat-8 carries two sensors, the Operational Land Imager and the Thermal Infrared Sensor TIRS. There are 11 bands in total. Bands 1–7 and 9–11 have a spatial resolution of 30 m, and Band 8 is a panchromatic band with a resolution of 15 m, all of which were obtained from the United States Geological Survey (USGS) website (http://landsat.visibleearth.nasa.gov/). The satellite can achieve global coverage every 16 days. The OLI land imager has nine bands and an image width of 185 × 185 km. The Sentinel-2 multispectral imager covers 13 spectral segments (443–2190 nm) with a width of 290 km. The spatial resolution is 10 m (four visible spectral segments and one near-infrared spectral segment), 20 m (six red edge spectral segments and short-wave infrared spectral segments), and 60 m (three atmospheric correction spectral segments). The data were downloaded using GEE, limiting cloud cover to less than 10% [18,19].

**Table 1:** Remote sensing image data.

Imaging data	Sensor	Resolution	Spectral bands
July 2020	Landsat OLI	30	B1(blue), B2(Geen), B3(Red), B4(NIR), B5(SWIR1), B6(SWIR2)
August 2020	Sentinel MSI	20	B1(blue), B2(Geen), B3(Red), B4(Red Edge), B5(Red Edge), B6(Red Edge), B7(NIR), B5(SWIR1), B6(SWIR2)

## 3. Research methods

### 3.1 Derivative processing of remote sensing satellite data

The derivative algorithm is beneficial to reduce image noise and improve the spectral sensitivity of ground objects [23,24]. The integer derivative approach mainly includes first-, second-, and third-order derivatives. The first derivative (FD) is the slope of the spectral curve, and the second derivative (SD) is the change rate of the slope of the spectral curve. The FD and SD transformation of the data can magnify the differences in the spectral information, facilitate the reduction of spectral noise, and further obtain useful spectral efficiency information [22]. In this study, IDL programming was used to realize the derivative processing of image data. The FD, SD, and TD transformations were performed on seven bands of OLI data and nine bands of Sentinel MSI data.

### 3.2 Relationship between spectral parameters and soil salinity

We examined previous research results and found that most scholars used single-band modeling for multi-spectral monitoring of soil salinization. Therefore, we first verified the universality of a single-band model in monitoring soil salt content in the Bosten Lake watershed (Table 2), and analyzed the correlation between multi-spectral single band and measured salt content based on linear regression.

**Table 2:** The relationship between Landsat image data OLI, Sentinel-2data and soil salt.

Landsat OLI Band	R^2^	References	Sentinel MSI Band	R^2^	References
Blue	0.78	Meng [25]	Blue	0.291	Mao [26]
Green	0.77	Irons [27]	Geen	0.313	Mao [26]
Red	0.86	Meng [16]	Red	0.321	Mao [26]
NIR	0.91	Irons [27]	Vegetation Red Edge	0.368	Mao [26]
SWIR1	0.52	Meng [25]	Vegetation Red Edge	0.421	Mao [26]
SWIR2	0.87	Lu [28]	Vegetation Red Edge	0.379	Mao [26]
SWIR2	0.67	Chander [29]	NIR	-0.380	Mao [26]
			SWIR 1 and 2	0.473/0.447	Mao [26]

This study summarized previous research results on hyperspectral soil salinization monitoring, selected widely used spectral indexes (Table 3) and applied linear regression to study the correlation between each spectral parameter and the measured salt content in the Bosten Lake watershed.

**Table 3:** Relationship between soil salt content and spectral index.

Spectral parameters	Abbreviation	Formula	R^2^	References
Simple ratio water index	SI-T	Red/Green*100%	0.71	Wang [30]
Normalized difference water index	NDSI	(Red-NIR)/ (Red+NIR)	0.57	Wang [30]
The ratio index	SI	Blue/Red	0.61	Liang, [31]

### 3.3 Construction of a spectral index for soil salinity estimation

The variable factors of remote sensing data were extracted to improve the sensitivity of remote sensing data to soil salinity information [6,29] and analyze the relationship between soil spectral index and soil salt content in the Bosten Lake watershed. Based on the correlation between the spectral difference index (DI), spectral ratio index (RI), and spectral normalization index (NDI) and soil salt content, the best combination bands for soil salt content estimation and inversion were selected [32].

$$DI(R_{i},R_{j})=R_{i}-R_{j}$$ (1)

$$NDI(R_{i},R_{j})=\frac{R_{i}-R_{j}}{R_{i}+R_{j}}$$ (2)

$$RI(R_{i},R_{j})=\frac{R_{i}}{R_{j}}$$ (3)

where *R*_*i*_ and *R*_*j*_ are arbitrary two waves in the band range of 350–2500 nm, and *R*_*i*_ and *R*_*j*_ are the reflectance of any two bands in the band range of 350–2500 nm. The optimum spectral parameters for estimating soil salt content were selected based on an analysis of the quantitative relationship between the spectral parameters and the soil salt content.

### 3.4 Grid search-support vector machines (GS-SVM)

The grid search (GS) method is an exhaustive search method for specifying parameter values [33]. The optimal learning algorithm is obtained by optimizing the parameters of the estimated function through cross validation. Support vector machines are a type of machine learning technology based on the principle of structural risk minimization. This approach can solve the problems of small sample, nonlinear, high dimension and local minimum well, and has excellent prediction and generalization ability. The penalty factor C and the kernel function parameter σ in an SVM directly affect the prediction accuracy of the model. In this study, the GS method was used to optimize SVM parameters. Of all the alternative parameters, the method iterates through every possibility and the one that performs best is selected as the final result. In the SVM model, the general parameters are C and g. Of these, C is the penalty coefficient, representing the tolerance for error. The higher the value of C, the less error is tolerated and the easier it is to overfit, whereas the smaller the value of C, the lower its fit. If C is too large or too small, the generalization capability becomes worse. Gamma is a built-in parameter of the RBF function selected as the kernel. It implicitly determines the distribution of data after mapping to the new feature space. The larger the value of gamma, the fewer support vectors, whereas the smaller the value of gamma, the more support vectors there are. The number of support vectors affects the speed of training and prediction [33].

### 3.5 Statistical analysis and model verification

In this study, the fitting coefficient *R*^2^, the standard deviation SD, the relative analysis RPD, and the root mean square error RMSE were selected to verify the accuracy and reliability of the model construction [34–36]. The smaller the value of RMSE, the more stable the prediction, estimation, and analysis ability of the model. The closer *R*^2^ is to 1, the higher the accuracy of the model. The RPD refers to relative analysis error. RPD < 1.4 indicates that the model is unreliable, whereas 1.4 < RPD < 2 indicates that the model has a general accuracy. RPD > 2 indicates that the model has a high prediction ability.

## 4 Results and analysis

### 4.1 Analysis of soil spectral characteristics with different salt contents in the study area

Table 4 shows the statistical results of salt content in soil samples of wetland surface in the study area. The maximum value of soil salt content in the Bosten Lake watershed was 11.8 g/kg. The minimum value was 0.41 g/kg, and the average value was 4.77 g/kg. In the Bosten Lake watershed, the climate was dry in the studied season, and evaporation was intense. The water in the soil increases strongly, and the loss of groundwater was mainly through evaporation, which promotes the accumulation of salt to the soil surface and causes the general accumulation of salt in wetland soil. In August, the water level of the watershed decreased, and evaporation on the surface salt was intense, leading to a maximum surface salt content as high as 57.8 g/kg. The minimum salt content was mainly distributed in the Kaidu River mountain area, which has the highest altitude of the whole Bosten Lake watershed, with low salt content and a great difference in soil salt content through the watershed.

**Table 4:** The soil salinity data statistics in Bosten Lake watershed.

	Dataset	Max	Min	Mean	Standard deviation
Dateset	43	11.8	0.79	5.22	4.77

**Fig 2:**
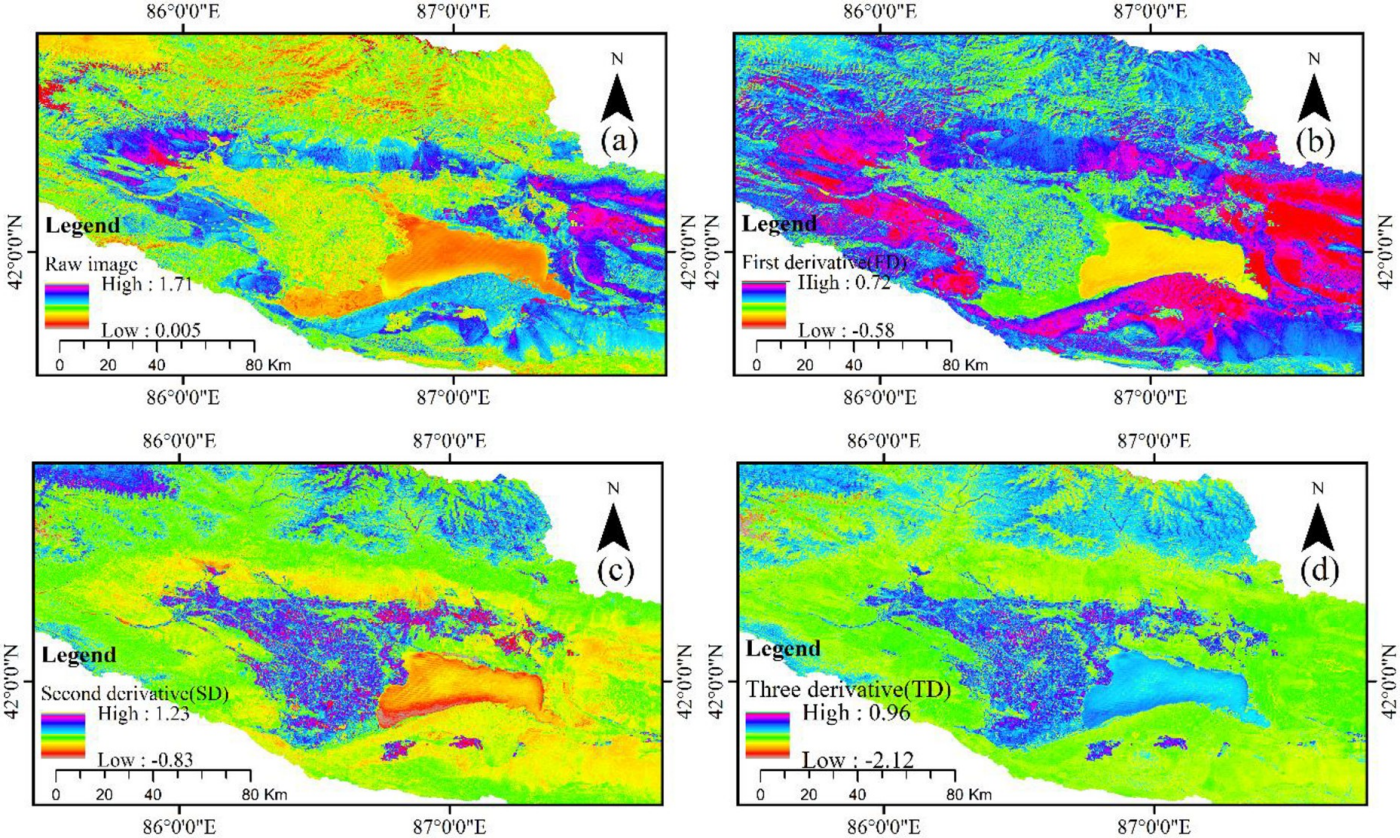
OLI data of the raw image (R), first derivative (FD), second derivative (SD), and third derivative (TD).

**Fig 3:**
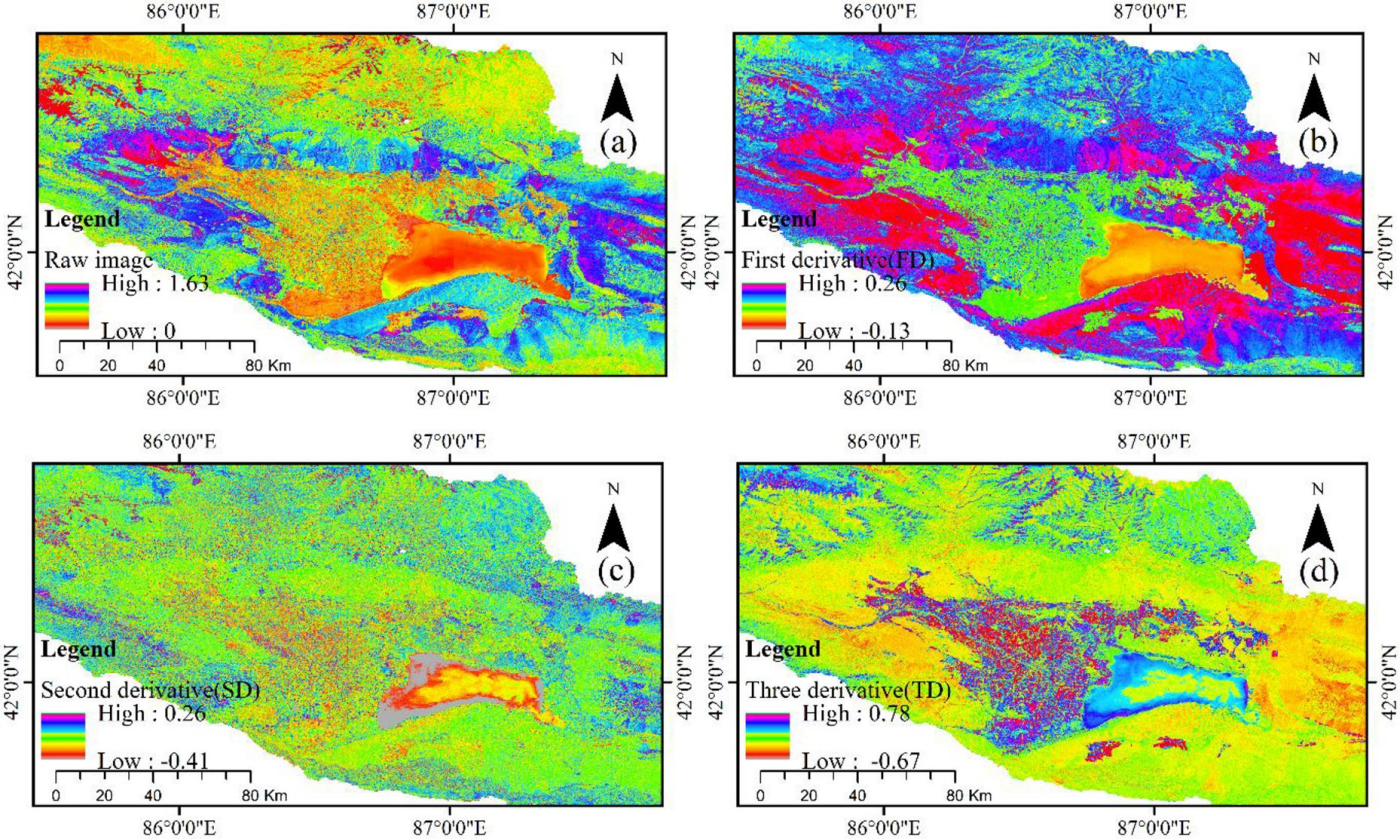
MSI data of the raw image (R), first derivative (FD), second derivative (SD), and third derivative (TD).

Figs 2 and 3 show the green band visual information processed by FD, SD, and TD algorithms on MSI data and OLI data, which is used to express the separation degree of regional vegetation from other substrates.

A derivative algorithm with OLI data of original data was found, and FD and SD were used to distinguish between vegetation and water bodies. The water bodies in the raw data and lakeside area of arable land had similar spectral characteristics; however, surface analysis was very good in the FD data, but the TD and SD favored mountain, and lake plain and mountain spectrum differentiation was very clear, especially in the TD data. As a result of the difference in spatial resolution between OLI data and MSI data, spatial scale effect and mixed pixels result in a great difference in the spectral differential algorithm effect between the two datasets. We found that the spectral characteristics of MSI data and OLI data were similar to those of FD. However, SD and TD differed significantly from OLI data in spectral characteristics, which not only facilitates the separation of land and water, but can also show the depth of the lake through spectral expression. For the expression of other ground object information, TD data of MSI was the best among all data and could distinguish grassland, cultivated land and water bodies. Moreover, the spectral difference of soil information of different attributes was clear, especially in the south of Bosten Lake, and the information of sand dunes and saline land was also very clear.

### 4.2 Analysis of the relationship between soil salt content and spectral characteristic value in the study area

When the single-band spectral parameters (Table 2) were applied to the monitoring of soil salinity in Bosten Lake watershed, it was found that the application of the single-band model of original image data in the estimation of soil salinization in Bosten Lake watershed was not universal. Moreover, the RPD values were all less than 1.4 (Table 5), indicating that the model was not reliable. Therefore, the single band model of original data could not meet the monitoring and inversion of soil salinity in the Bosten Lake watershed.

**Table 5:** Relationship between soil salt content and spectral characteristics of MSI and OLI data.

Dataset	Band (X)	Formula	R^2^	RMSE	RPD
MSI	Blue (B_1_)	Y = 29.41x+1.41	0.29	4.17	1.07
	Geen (B_2_)	Y = 26.43x+0.68	0.29	4.11	1.08
	Red (B_3_)	Y = 22.98x+0.62	0.28	4.11	1.09
	Red Edge (B_4_)	Y = 26.15x-0.63	0.29	4.19	1.06
	Red Edge (B_5_)	Y = 21.77x-0.19	0.11	4.49	0.99
	Red Edge (B_6_)	Y = 13.94x+1.61	0.07	5.21	0.85
	NIR (B_7_)	Y = 8.84x+2.87	0.09	5.01	0.89
	SWIR1 (B_8_)	Y = 18.16x-0.11	0.26	3.98	1.12
	SWIR2 (B_9_)	Y = 15.32x+1.26	0.26	3.99	1.12
OLI	Blue (B_1_)	Y = 55.3x-1.35	0.29	4.28	1.04
	Green (B_2_)	Y = 43.8x-2.15	0.32	4.15	1.07
	Red (B_3_)	Y = 32.48x-1.27	0.29	4.21	1.06
	NIR (B_4_)	Y = 34.14x-3.86	0.13	4.95	0.97
	SWIR1 (B_5_)	Y = 23.44x-0.72	0.31	3.85	1.16
	SWIR2 (B_6_)	Y = 26.48x-2.41	0.39	3.72	1.20

When the optimal spectral index (Table 3) monitoring of soil salt content was applied to the monitoring of soil salt content in the Bosten Lake watershed, it was found that the optimal model was not universal (Table 6), and most models could not meet the monitoring needs for soil salt content in this arid area.

**Table 6:** Relationship between soil salt content and measured spectral index in Bosten Lake Watershed.

	Spectral parameters (X)	Formula	R^2^	RMSE	RPD
MSI	SI-T	Y = -1.79x+97.68	0.34	3.85	1.23
	NDSI	Y = 0.04x-0.036	0.33	3.96	1.20
	SI	Y = 0.03x+0.73	0.29	4.05	1.17
OLI	SI-T	Y = -1.71x+92.35	0.26	4.12	1.15
	NDSI	Y = 0.05x-0.24	0.33	3.96	1.20
	SI	Y = 0.03x+0.88	0.29	4.05	1.17

### 4.3 Construction of remote sensing index for estimation of soil salinity in the study area

Based on Formulas (1–3), a spectral index suitable for the Bosten Lake watershed was constructed using the relationship between the ratio, difference, and normalized index of the original spectra of two bands common in Landsat OLI data and the soil salt content.

**Fig 4:**
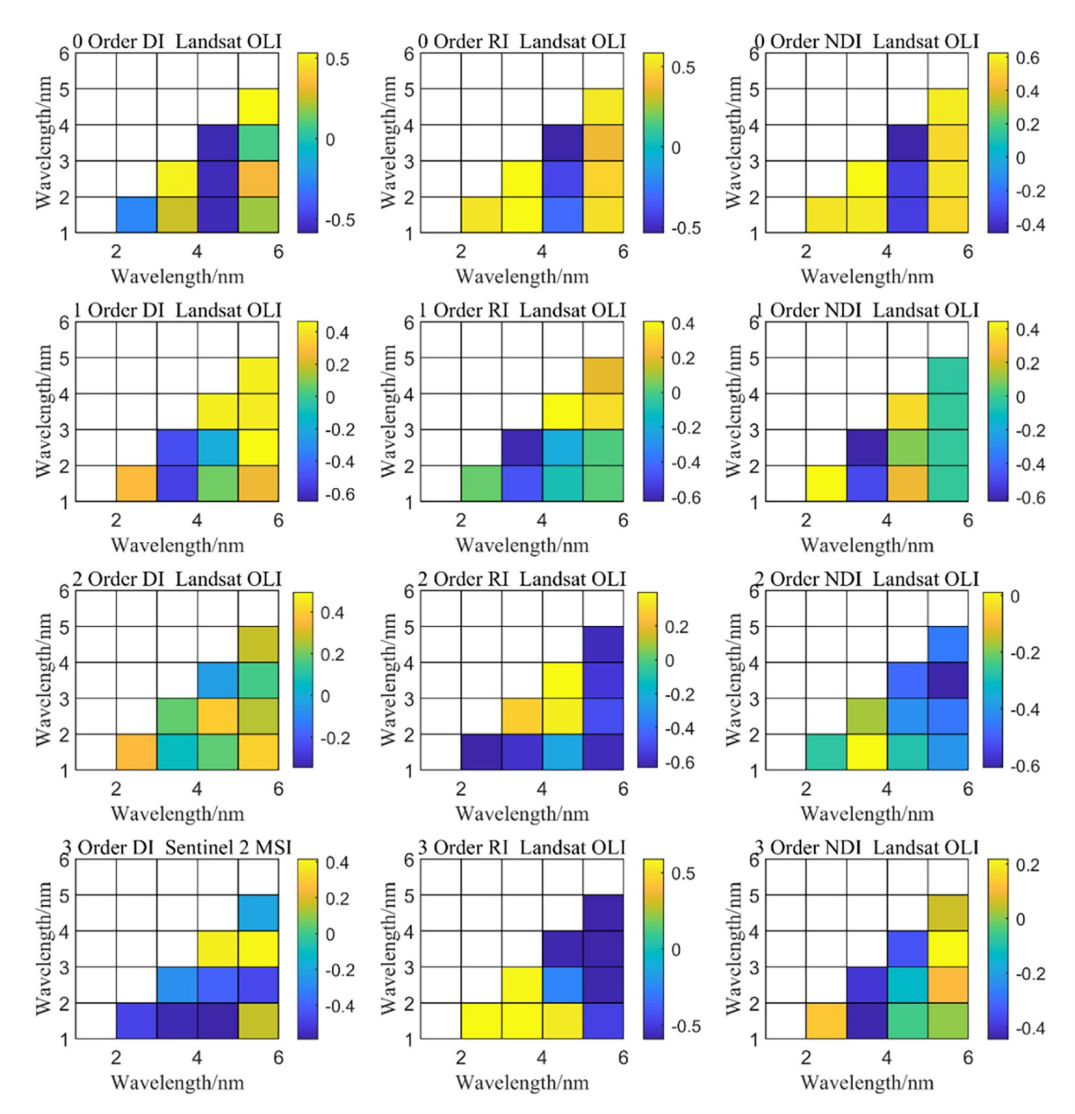
Spectral DI, NDI, and RI indices of soil salinization based on OLI data.

Fig 4 shows that the optimal combination of normalized spectral data and soil salinity in the original data was normalized [(B_6_-B_5_)/(B_6_+B_5_)] index (*R*^2^ = 0.41), followed by difference (B_6_-B_5_) index (*R*^2^ = 0.26) and the ratio index (B_3_/B_2_)(*R*^2^ = 0.28). In the SD, the optimal band combination was the ratio index (B_6_/B_1_) (*R*^2^ = 0.42). For the difference index (B_6_-B_1_), *R*^2^ was 0.24 and for the normalized index [(B_4_-B_6_)/(B_4_+B_6_)], *R*^2^ was 0.42. In the TD, the optimal band combination was the ratio index (B_6_/B_5_) with *R*^2^ of 0.37, the difference index (B_5_-B_1_) had an *R*^2^ of 0.25, and the normalized [(B_4_-B_6_)/(B_4_+B_6_)] index had *R*^2^ of 0.18. Therefore, we constructed a remote sensing index for monitoring surface salinization in the Bosten Lake watershed based on OLI data.

**Fig 5:**
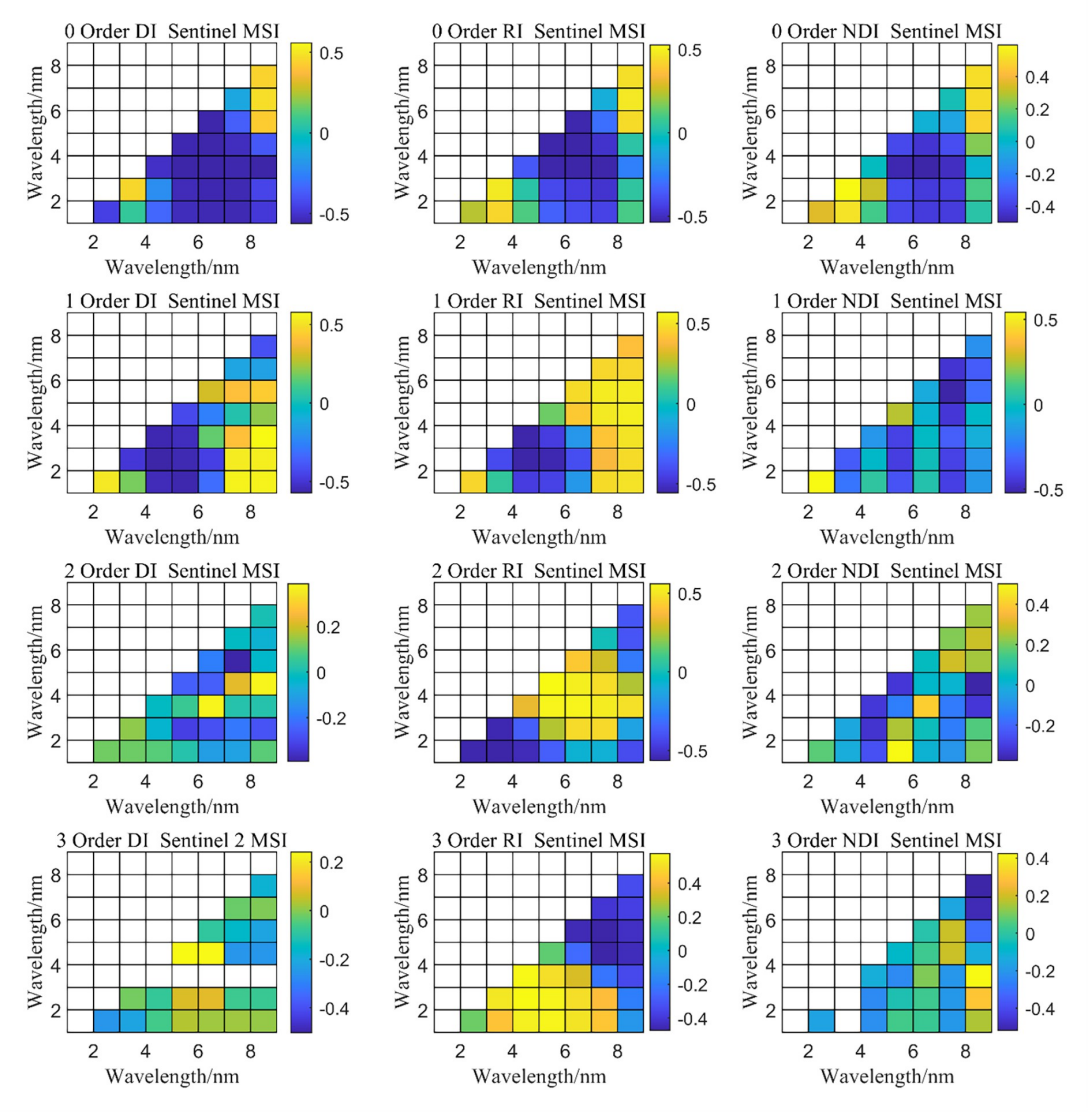
Spectral DI, NDI and RI indices of soil salinization based on MSI data.

Fig 5 shows the relationship between spectral data of original data and the soil salt content. The *R*^2^ of ratio (B_9_/B_7_) index was 0.33, that of difference (B_9_-B_7_) index was 0.32, and that of normalized index [(B_9_-B_7_)/(B_9_+B_7_)] was 0.29. Fig 5 shows the relationship between spectral data of the FD and the soil salt content. The *R*^2^ of ratio index (B_9_/B_7_), difference index (B_1_-B_9_), and normalization index [(B_2_-B_1_)/(B_2_+B_1_)] was 0.29. The relationship between the SD spectral data and soil salt content was also analyzed. The *R*^2^ of the ratio index B_9_/B_4_), difference index (B_9_-B_5_), and normalization index [(B_4_-B_7_)/(B_4_+B_7_)] was 0.27, 0.15, and 0.31, respectively. The relationship between the spectral data of the TD and soil salt content showed that the *R*^2^ of ratio index (B_7_/B_5_) was 0.28, that of difference (B_7_-B_3_) was 0.15, and that of normalization [(B_3_-B_9_)/(B_3_+B_9_)] was 0.32.

### 4.4 Construction of a soil salinity estimation model based on the GS-SVM model in the Bosten Lake watershed

In this study, the structure of the GS-SVM regression model was designed using MATLAB2018. The DI, RI, and NDI data of 43 pixels (OR, FD, SD and TD) in remote sensing images and the DI, RI, and NDI of the three transformations of measured spectral data (OR, FD, SD and TD) were used to select 30 groups of soil salt content. Finally, 13 groups of DI, RI, and NDI and soil salt content were randomly reserved for verification. The penalty factor C and the kernel function parameter σ in the GS-SVM model directly affect the prediction accuracy of the model. The GS method was selected in this paper for GS-SVM parameter optimization, and the most input parameters are shown in Table 7.

**Table 7:** Optimal parameter selection of SVM based on GS method.

Data source	Data transformation	Parameters	Best c	g	CV Accuracy
MSI	Raw	RI、DI、NDI	0.25	11.31	96.62%
	FD	RI、DI、NDI	1.41	8.00	93.25%
	SD	RI、DI、NDI	0.35	1.41	66.29%
	TD	RI、DI、NDI	0.25	11.31	96.62%
OLI	Raw	RI、DI、NDI	1.41	8.00	93.25%
	FD	RI、DI、NDI	0.25	11.31	96.62%
	SD	RI、DI、NDI	1.41	8.00	93.25%
	TD	RI、DI、NDI	0.35	1.41	66.29%

**Fig 6:**
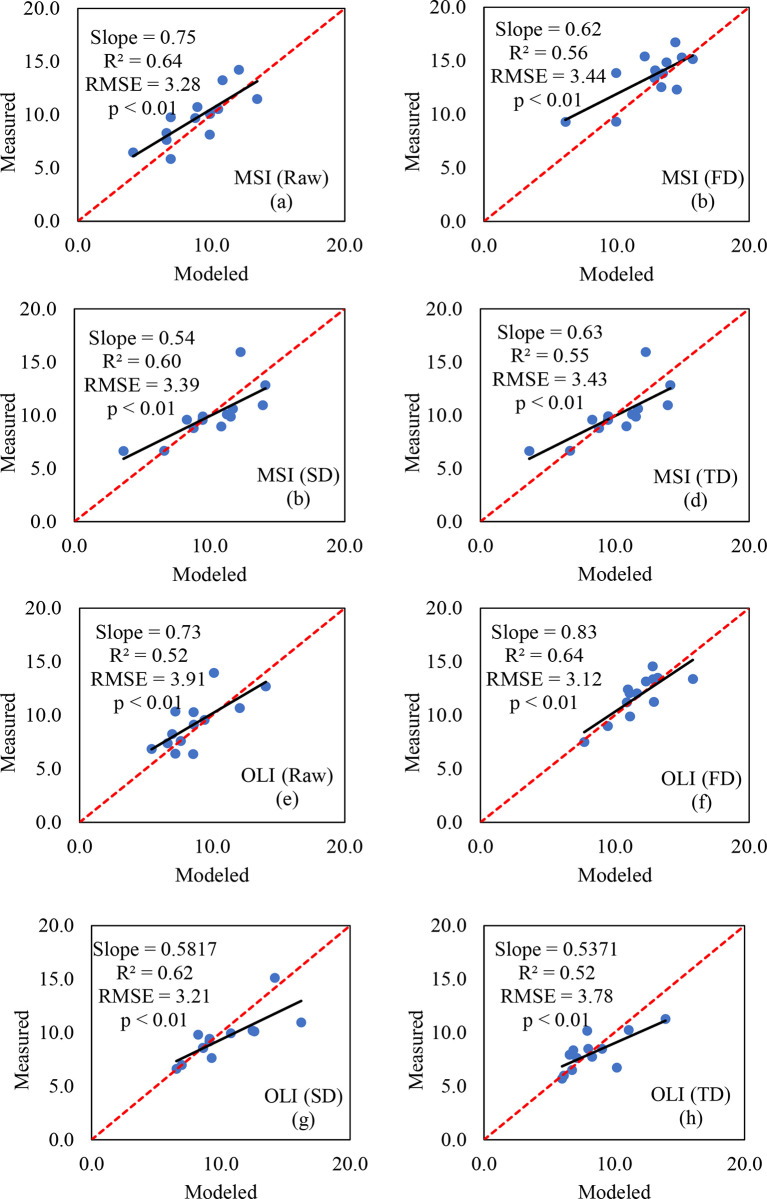
Scatter plot of measured and predicted soil salinity in GS-SVM models.

The new spectral index has a high simulation accuracy for the SVM model verification of soil salinity in the Bosten Lake watershed. Table 8 and Fig 6 shows that the *R*^2^ of the predicted value of the inversion model and the measured value was greater than 0.52, and the RMSE was low. The original data of MSI data had the best estimation accuracy, *R*^2^ is 0.64, RMSE was 3.28, RPD is 1.33, and the model was stable. In OLI data, the optimal *R*^2^ of the FD data was 0.64, RMSE was 3.12, and RPD was 1.64. We found that the spatial resolution of remote sensing data does not affect the inversion precision of soil salinization; the 20 m spatial resolution of the inversion precision of MSI data close to 30 m OLI data of spatial resolution. However, it should be noted that this is a relatively short time series, and MSI data that will be used in long time series of the OLI FD data time series, the salinization in the study area mapping.

**Table 8:** Validation of SVM model for estimating soil salinity in Bosten Lake Watershed.

Data source	Order	Verification Dataset	Parameters	R^2^	RMSE	SD	RPD
MSI	Raw	13	RI、DI、NDI	0.64	3.28	4.36	1.33
	FD	13	RI、DI、NDI	0.56	3.44	4.08	1.18
	SD	13	RI、DI、NDI	0.60	3.39	4.64	1.37
	TD	13	RI、DI、NDI	0.55	3.43	4.07	1.15
OLI	Raw	13	RI、DI、NDI	0.52	3.91	4.38	1.12
	FD	13	RI、DI、NDI	0.64	3.12	5.13	1.64
	SD	13	RI、DI、NDI	0.62	3.21	4.46	1.39
	TD	13	RI、DI、NDI	0.52	3.78	4.36	1.11

### 4.5 Analysis of remote sensing inversion results of soil salinity

**Fig 7:**
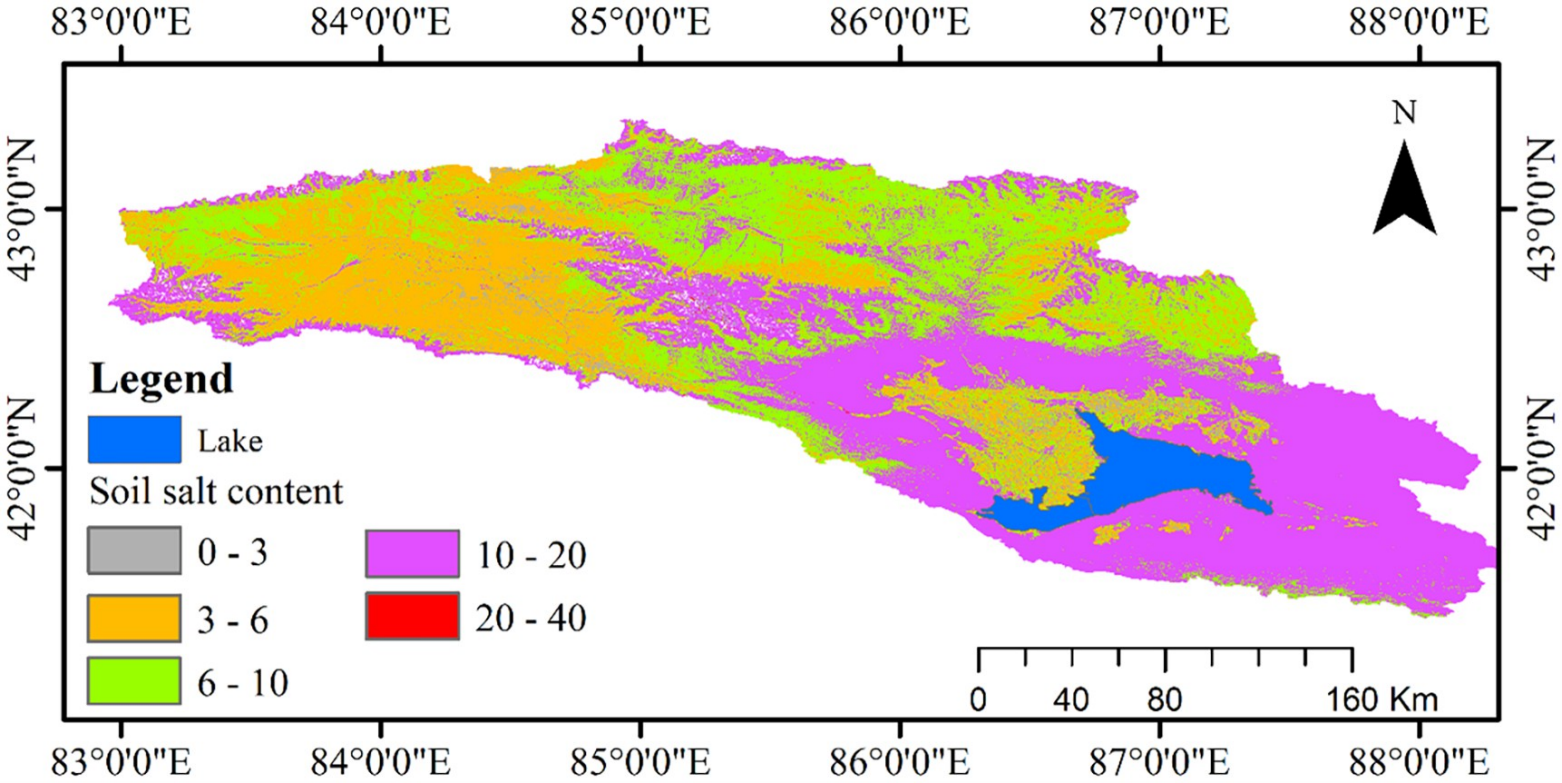
Map of soil salt content based on the SVM model.

The spatial pattern chart of each salinization level was divided according to the classification standard of the soil salinization level (Fig 7) [36]. Fig 7 shows that each salinization level was clearly distinguished. In this study, remote sensing inversion of soil salinity in the Bosten Lake watershed was conducted based on the FD model of Landsat OLI data, and manual classification was conducted in ArcGIS software according to classification. We found that the non-salinized area accounted for 3.73%, mild salinization accounted for 29.44%, moderate salinization accounted for 25.11%, severe salinization accounted for 39.30%, and salinization accounted for 2.42% in the Bosten Lake watershed.

Terrain and climate are the main controls on soil salinization. The Bosten Lake watershed is located in the Yanqi watershed, and the terrain is high in the north and low in the south. Between the piedmont and Bosten Lake, there is a piedmont alluvial incline plain, the Kaidu River Delta and the Bosten Lake watershed. The flat terrain on the west and east sides of Bosten Lake is more susceptible to the change of lake water level, whereas the south and north sides are less affected by the lake water level because of the higher terrain. The Yanqi watershed is mainly dominated by farmland and the flat terrain is seriously salinized, and this represents the main area of severe salinization in the watershed. The Kaidu River watershed in the northwest is mainly salinized because of its high altitude and animal husbandry. The soil in other watersheds is mainly salinized because of high altitude and shows a moderate level of salinization.

## 5 Discussion

### 5.1 Derivative algorithm is useful for remote sensing data denoising

In this study, an image derivative algorithm was mainly used for data processing. The image derivative algorithm can magnify the difference of spectral information and facilitate the extraction of effective spectral information. We attempted to conduct derivative processing on the image data and hyperspectral data. The FD was the slope of the spectral curve, and the SD was the change rate of the slope of the spectral curve [36]. The FD and SD magnify the spectral difference and improve the accuracy of the model; however, the integer 1, 2, 3, derivative, and the original spectrum curve difference is bigger, which can lead to missing information and limited accuracy of ascension. Moreover, if you consider it introduced the fractional order differential spectral domain, it may further refine the difference between the spectral data, to highlight its effective information and improve the monitoring model accuracy. In this study, a soil remote sensing salinization spectral index was constructed for soil salinity inversion, and multi-band remote sensing data were selected as variable factors. It was found that the sensitivity of the combined operation for different bands was significantly improved compared with the single band model, highlighting the advantage of the application of band combination and further laying a foundation for the multi-spectral inversion of soil salinity. However, further research is needed to determine whether this research method is applicable to other areas in the arid zone, particularly to determine whether other data sources have better inversion effects.

### 5.2 The GS-SVM model is a potential remote sensing model for soil salinity

This study examined the application and potential of previous research (Tables 2 and 3) results in remote sensing estimation model of SCC in Bosten Lake watershed in an arid area, and found that previous research results could not meet the monitoring needs. Therefore, we proposed an optimal and universal soil salinization monitoring model suitable for estimating the soil salt content in arid region of Central Asia. First, combined with Landsat OLI data and Sentinel MSI data, and through image differential processing of the image data, a soil salinity monitoring model suitable for the Bosten Lake watershed was constructed. To evaluate the accuracy of the model, DI, RI and NDI of OLI image second-order data were used as input data to compare the estimation accuracy of GS-SVM, SVM, Partial Least Squares Regression (PLSR) and Linear regression (LR) models. The results are shown in Table 9. Therefore, Multi-model comparison shows that GS-SVM model has higher estimation ability and reliability.

**Table 9:** Comparison of the accuracy of estimating soil salt content by different models.

Order	Verification Dataset	Parameters	R^2^	RMSE	SD	RPD
GS-SVM	13	RI、DI、NDI	0.64	3.12	5.13	1.64
SVM	13	RI、DI、NDI	0.61	3.94	4.03	1.02
PLSR	13	RI、DI、NDI	0.56	4.18	4.09	0.97
LR	13	RI、DI、NDI	0.49	4.22	4.03	0.95

## 6 Conclusion

We conducted remote sensing inversion research on soil salinity in Bosten Lake watershed, a typical watershed in an arid area. Sentinel MSI and Landsat OLI data were combined with measured soil salinity data, and the optimal combination bands were selected through the selection of characteristic bands to create a GS-SVM inversion model of soil salinity. The results are listed as following:

1. The results of previous studies could not meet the monitoring requirements of the study area (*R*^2^ < 0.3).
2. A GS-SVM soil salinity monitoring model was established based on the optimal DI, RI, and NDI remote sensing indexes of Bosten Lake watershed. After model verification, it was found that the optimal model of image data was the Landsat OLI first-derivative model with *R*^2^ of 0.64, RMSE of 3.12 and RPD of 1.64, indicating that the prediction ability of the model was high.
3. We used the first-order derivative model of Landsat OLI data to map the soil salt content in the Bosten Lake watershed, and found that soil salt content in most areas of the study area was between 10 and 20 g/kg, indicating severe salinization. This study not only shows the distribution characteristics of salinization in Bosten Lake watershed, but also provides a scientific basis for soil salinization monitoring in Central Asia, laying a foundation for soil salinization monitoring in arid areas.

## Supporting information

S1 File All relevant data are within the paper and its supporting information files. Please inform the authors if data are being used. The Sentinel-2 and Landsat data (Figs 2 and 3) are freely available at http://landsat.visibleearth.nasa.gov/.
(ZIP)
