# Supplementary material for: Estimation of soil salt content in the Bosten Lake watershed, Northwest China based on a support vector machine model and optimal spectral indices
Source: PLoS One. 2023 Feb 24;18(2):e0273738. doi: 10.1371/journal.pone.0273738 (PMC9955642; doi:10.1371/journal.pone.0273738)
Supplement: S1 File — Please inform the authors if data are being used. The Sentinel-2 and Landsat data (Figs 2 and 3) are freely available at http://landsat.visibleearth.nasa.gov/. (ZIP) [file pone.0273738.s001.zip › Supplementary Materials/Figure 3.docx]

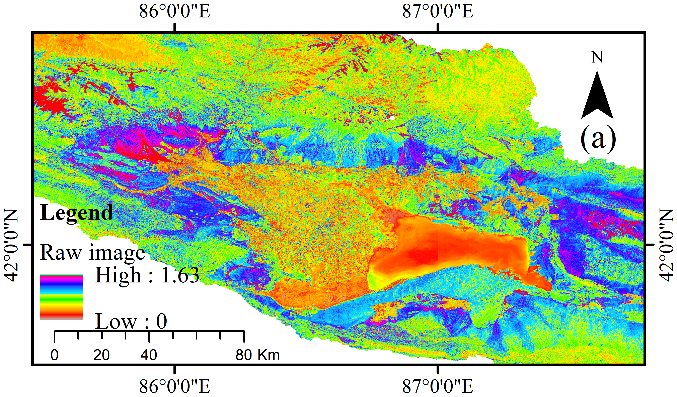

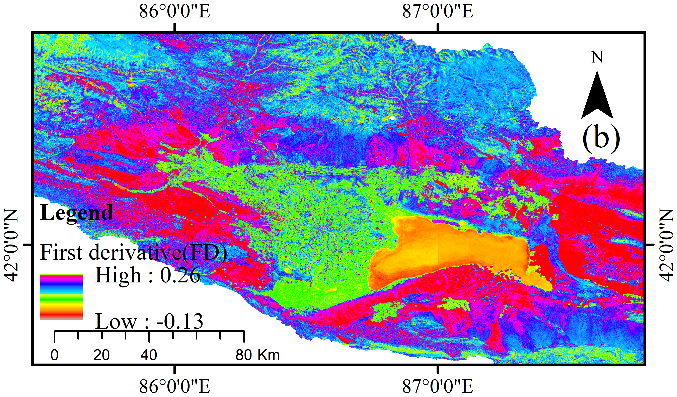


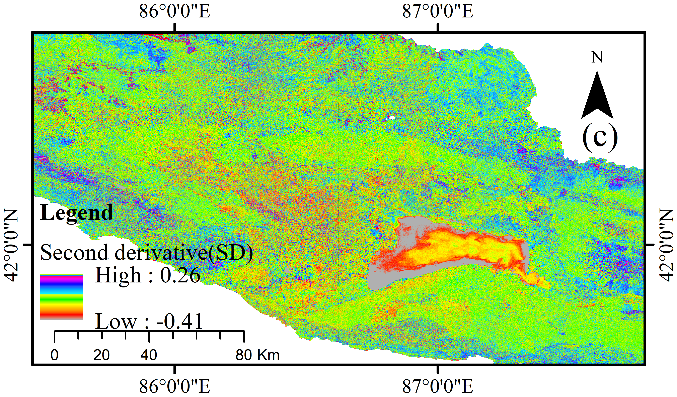

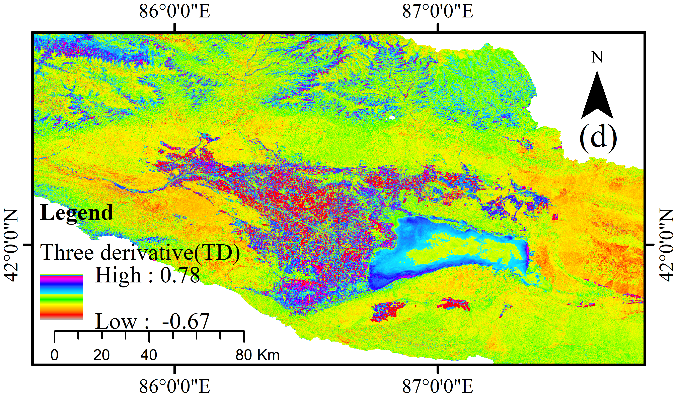


Figure 3 MSI data of the raw image (R), first derivative (FD), second derivative (SD), and third derivative (TD)
