# Supplementary material for: Estimation of soil salt content in the Bosten Lake watershed, Northwest China based on a support vector machine model and optimal spectral indices
Source: PLoS One. 2023 Feb 24;18(2):e0273738. doi: 10.1371/journal.pone.0273738 (PMC9955642; doi:10.1371/journal.pone.0273738)
Supplement: S1 File — Please inform the authors if data are being used. The Sentinel-2 and Landsat data (Figs 2 and 3) are freely available at http://landsat.visibleearth.nasa.gov/. (ZIP) [file pone.0273738.s001.zip › Supplementary Materials/Figure captions.docx]

Figure 1 Map of the Bosten Lake watershed

Figure 2 OLI data of the raw image (R), first derivative (FD), second derivative (SD), and third derivative (TD)

Figure 3 MSI data of the raw image (R), first derivative (FD), second derivative (SD), and third derivative (TD)

Figure 4. Spectral DI, NDI, and RI indices of soil salinization based on OLI data

Figure 5. Spectral DI, NDI and RI indices of soil salinization based on MSI data

Figure 6 Scatter plot of measured and predicted soil salinity in GS-SVM models

Figure 7. Map of soil salt content based on the SVM model
