# Supplementary material for: Estimation of soil salt content in the Bosten Lake watershed, Northwest China based on a support vector machine model and optimal spectral indices
Source: PLoS One. 2023 Feb 24;18(2):e0273738. doi: 10.1371/journal.pone.0273738 (PMC9955642; doi:10.1371/journal.pone.0273738)
Supplement: S1 File — Please inform the authors if data are being used. The Sentinel-2 and Landsat data (Figs 2 and 3) are freely available at http://landsat.visibleearth.nasa.gov/. (ZIP) [file pone.0273738.s001.zip › Supplementary Materials/Table 1.docx]

Table 1 Remote sensing image data

| Imaging data | Sensor | Resolution | Spectral bands |
| --- | --- | --- | --- |
| July 2020 | Landsat OLI | 30 | B1(blue), B2(Geen), B3(Red), B4(NIR), B5(SWIR1), B6(SWIR2) |
| August 2020 | Sentinel MSI | 20 | B1(blue), B2(Geen), B3(Red), B4(Red Edge), B5(Red Edge), B6(Red Edge), B7(NIR), B5(SWIR1), B6(SWIR2) |
