# Supplementary material for: Estimation of soil salt content in the Bosten Lake watershed, Northwest China based on a support vector machine model and optimal spectral indices
Source: PLoS One. 2023 Feb 24;18(2):e0273738. doi: 10.1371/journal.pone.0273738 (PMC9955642; doi:10.1371/journal.pone.0273738)
Supplement: S1 File — Please inform the authors if data are being used. The Sentinel-2 and Landsat data (Figs 2 and 3) are freely available at http://landsat.visibleearth.nasa.gov/. (ZIP) [file pone.0273738.s001.zip › Supplementary Materials/Table 2.docx]

Table 2 The relationship between Landsat image data OLI, Sentinel-2data and soil salt

| Landsat OLI Band | R^2^ | References | Sentinel MSI Band | R^2^ | References |
| --- | --- | --- | --- | --- | --- |
| Blue | 0.78 | Meng^[26]^ | Blue | 0.291 | Mao^[25]^ |
| Green | 0.77 | Irons^[27]^ | Geen | 0.313 | Mao ^[25]^ |
| Red | 0.86 | Meng^[16]^ | Red | 0.321 | Mao ^[25]^ |
| NIR | 0.91 | Irons^[27]^ | Vegetation Red Edge | 0.368 | Mao ^[25]^ |
| SWIR1 | 0.52 | Meng^[26]^ | Vegetation Red Edge | 0.421 | Mao ^[25]^ |
| SWIR2 | 0.87 | Lu^[28]^ | Vegetation Red Edge | 0.379 | Mao ^[25]^ |
| SWIR2 | 0.67 | Chander^[29]^ | NIR | -0.380 | Mao ^[25]^ |
|  |  |  | SWIR 1 and 2 | 0.473/0.447 | Mao ^[25]^ |
