# Supplementary material for: Estimation of soil salt content in the Bosten Lake watershed, Northwest China based on a support vector machine model and optimal spectral indices
Source: PLoS One. 2023 Feb 24;18(2):e0273738. doi: 10.1371/journal.pone.0273738 (PMC9955642; doi:10.1371/journal.pone.0273738)
Supplement: S1 File — Please inform the authors if data are being used. The Sentinel-2 and Landsat data (Figs 2 and 3) are freely available at http://landsat.visibleearth.nasa.gov/. (ZIP) [file pone.0273738.s001.zip › Supplementary Materials/Table 3.docx]

Table 3 Relationship between soil salt content and spectral index

| Spectral parameters | Abbreviation | Formula | R^2^ | References |
| --- | --- | --- | --- | --- |
| Simple ratio water index | SI-T | Red/Green*100% | 0.71 | Wang ^[30]^ |
| Normalized difference water index | NDSI | (Red-NIR)/( Red+NIR) | 0.57 | Wang ^[30]^ |
| The ratio index | SI | Blue/Red | 0.61 | Liang,^[31]^ |
