# Supplementary material for: Estimation of soil salt content in the Bosten Lake watershed, Northwest China based on a support vector machine model and optimal spectral indices
Source: PLoS One. 2023 Feb 24;18(2):e0273738. doi: 10.1371/journal.pone.0273738 (PMC9955642; doi:10.1371/journal.pone.0273738)
Supplement: S1 File — Please inform the authors if data are being used. The Sentinel-2 and Landsat data (Figs 2 and 3) are freely available at http://landsat.visibleearth.nasa.gov/. (ZIP) [file pone.0273738.s001.zip › Supplementary Materials/Table captions.docx]

Table 1 Remote sensing image data

Table 2 The relationship between Landsat image data OLI, Sentinel-2data and soil salt

Table 3 Relationship between soil salt content and spectral index

Table 4 The soil salinity data statistics in Bosten Lake watershed

Table 5 Relationship between soil salt content and spectral characteristics of MSI and OLI data

Table 6 Relationship between soil salt content and measured spectral index in Bosten Lake Watershed

Table 7 Optimal parameter selection of SVM based on GS method

Table 8 Validation of SVM model for estimating soil salinity in Bosten Lake Watershed

Table 9 Comparison of the accuracy of estimating soil salt content by different models
